# Current Applications of Artificial Intelligence in Bariatric Surgery

**DOI:** 10.1007/s11695-022-06100-1

**Published:** 2022-05-26

**Authors:** Valentina Bellini, Marina Valente, Melania Turetti, Paolo Del Rio, Francesco Saturno, Massimo Maffezzoni, Elena Bignami

**Affiliations:** 1 Anesthesiology, Critical Care and Pain Medicine Division, Department of Medicine and Surgery, University of Parma, Viale Gramsci 14, 43126 Parma, Italy; 2 General Surgery Unit, Department of Medicine and Surgery, University of Parma, Viale Gramsci 14, 43126 Parma, Italy

**Keywords:** Artificial intelligence, Machine learning, Bariatric surgery, Perioperative medicine, Postoperative complications

## Abstract

The application of artificial intelligence technologies is growing in several fields of healthcare settings. The aim of this article is to review the current applications of artificial intelligence in bariatric surgery. We performed a review of the literature on Scopus, PubMed and Cochrane databases, screening all relevant studies published until September 2021, and finally including 36 articles. The use of machine learning algorithms in bariatric surgery is explored in all steps of the clinical pathway, from presurgical risk-assessment and intraoperative management to complications and outcomes prediction. The models showed remarkable results helping physicians in the decision-making process, thus improving the quality of care, and contributing to precision medicine. Several legal and ethical hurdles should be overcome before these methods can be used in common practice.

## Introduction

Artificial intelligence (AI) is the study of algorithms that give machines those abilities considered typical of human thinking, such as problem-solving, object and word recognition, inference of world states, and decision-making [1]. The capability of AI algorithms in the quick and accurate examination of large datasets, and in detection of correlations and patterns imperceptible for human mind, makes them particularly useful in healthcare setting. The relentless growth of AI implementation involves that it is of paramount importance to understand how these technologies can be used to deliver safer, more efficient, and more cost-effective care.

In the context of perioperative medicine, machine learning (ML) is having great success. Among the different classifications of ML available in literature, one of the most popular identifies three main categories: supervised, unsupervised, and reinforcement learning [2]. A supervised algorithm is a task-driven process where an algorithm is trained to predict a prespecified output; it requires a training dataset, to analyze and learn associations between an input and desired output, and a test dataset, used for the assessment of the algorithm performance on new data [2]. An unsupervised algorithm refers to algorithms that identify patterns or structure in an untagged dataset [3]. A reinforcement algorithm has to perform a certain task, learning from its mistakes and successes. Finally, a special note goes to deep learning, considered the most advanced class of ML algorithms, that uses multiple layers to progressively extract higher-level features from the raw input [4].

AI has different potential uses in modern medicine; in fact, it can implement and complement human intelligence by augmenting and democratizing it. AI-algorithms are increasingly used for genomics, imaging and diagnosis, risk stratification, and drug discovery [5]. It finds various applications in surgery [6, 7] and anesthesia [2, 8]. Furthermore, it can be applied for the evaluation and optimization of health conditions to better control and prevent chronic diseases in the idea of precision medicine [9].

The management of the patient candidate to bariatric surgery (BS) is an intricate topic. It requires evaluation by a multidisciplinary team consisting of internists, psychiatrists, general surgeons, and anesthesiologists. All physicians are involved in pre, intra, and postoperative evaluation which is challenging due to the complexity of the patients suffering from obesity [10, 11]. Compared to the non-obese patient, the process is elaborate, and risks-increased [12–14]. Management of comorbidities related to obesity, such as obstructive sleep apnea (OSA), diabetes, heart disease, hypertension, and gastroesophageal reflux disease, requires careful preoperative evaluation [15, 16]. BS can be more demanding than common general surgery, due to the intraoperative anesthesiologic control, which requires caution for the management of airways, ventilation, and hemodynamics [17–19]. Furthermore, the pharmacokinetic of drugs commonly used in anesthesia is different during bariatric surgical procedures and keeps being a thorny issue [20]. Pharmacological dosing must be carefully planned; weight-based drug dosing in patients with obesity can be defined on actual total body weight (TBW), ideal body weight (IBW), lean body weight (LBW), or adjusted body weight (AdjBW), depending on the specific drug [21, 22].

For these difficulties, we believe that the management of patients with obesity candidate for a bariatric procedure could obtain many advantages from the use of new data mining technologies. In this review, we analyzed the available literature on the current applications of AI to BS, evaluating its impact on each phase of the perioperative management, from presurgical assessment to postoperative period.

## Materials and Methods

We performed a narrative review of the literature on the Scopus, Pubmed, and Cochrane databases. All relevant studies published up to September 2021 were included. The search string included various combinations of “artificial intelligence,” “machine learning,” “obese patient,” “pathology,” “risk assessment,” and “bariatric surgery.” Papers concerning children, animals, and studies written in languages other than English were excluded. Articles of interest that were cited from the articles identified in the initial search were also included.

## Results

**Fig. 1:**
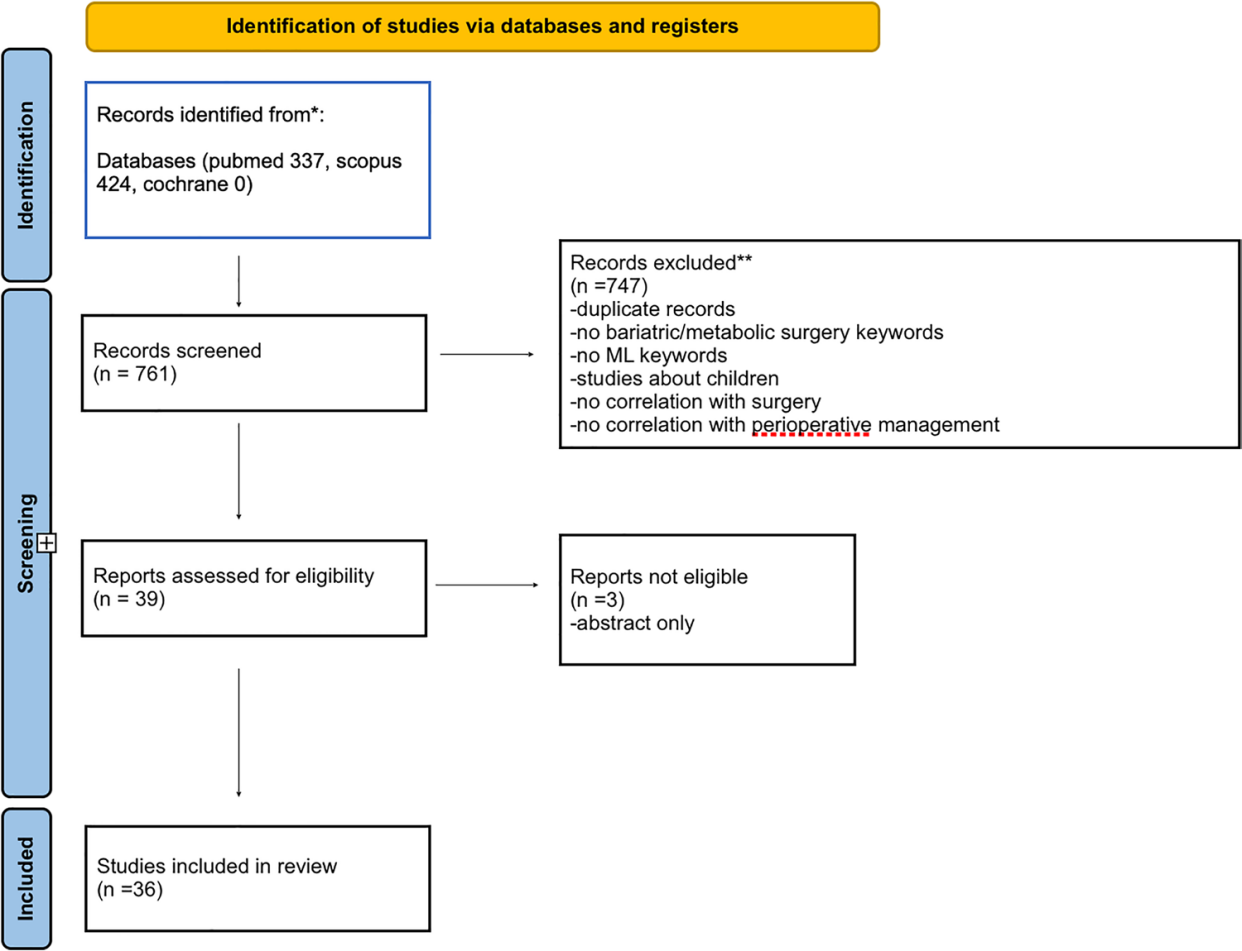
Article selection flow diagram

The literature search was conducted on 3 scientific databases (Pubmed, Scopus, and Cochrane) and produced 761 results. After the screening and removal of duplicates, 36 articles met the inclusion criteria and were finally included in the analysis (Fig. 1).

In detail, considering the design of the report, 28 studies were retrospective and 8 were prospective; 23 articles were about single-center research, while 13 were multicenter.

**Fig. 2:**
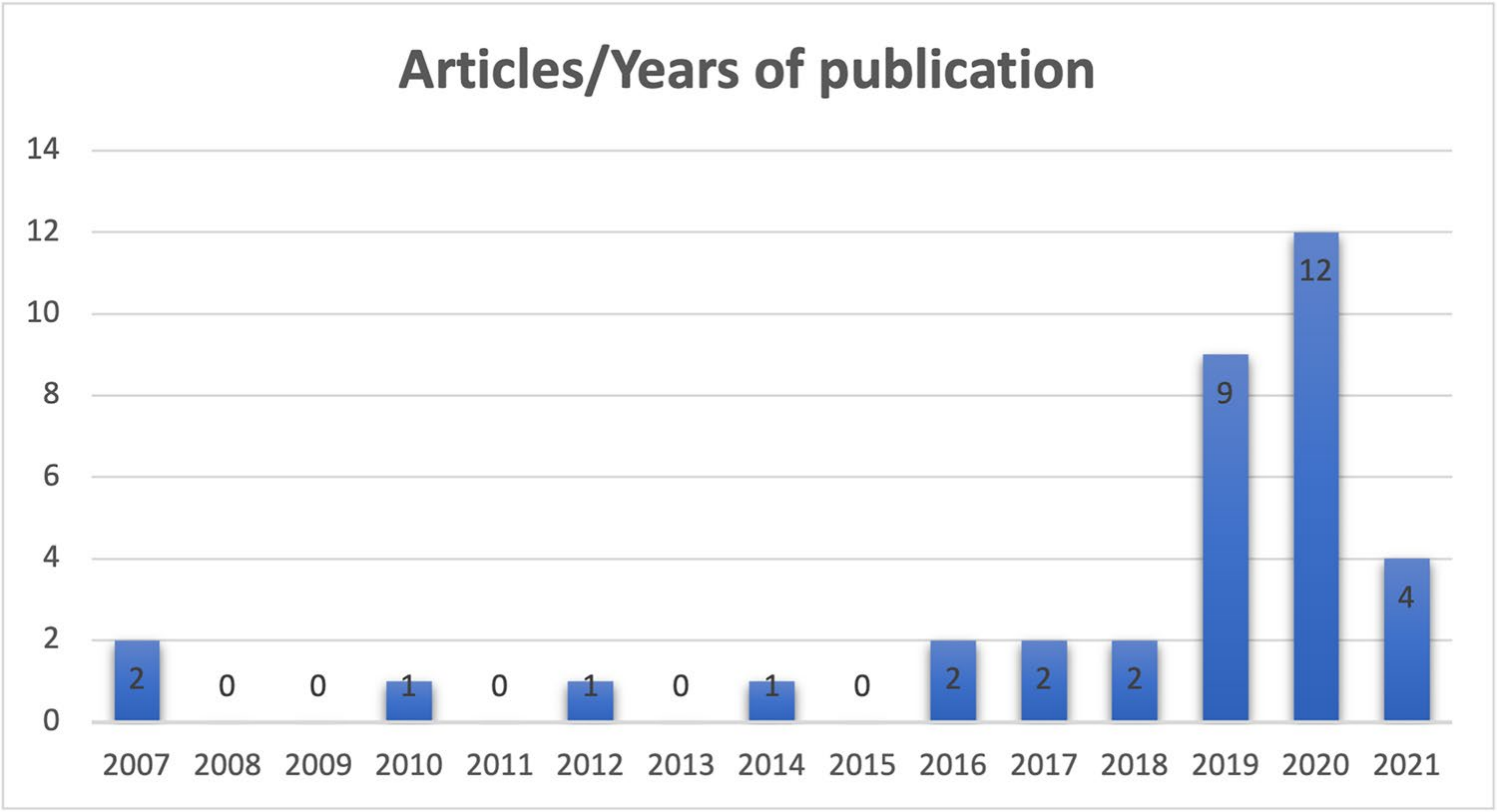
Temporal distribution of the articles included in our analysis according to the year of publication

Regarding the temporal distribution, the totality of the studies has been produced between 2001 and 2021, with a rising trend in last 3 years (Fig. 2).

**Fig. 3:**
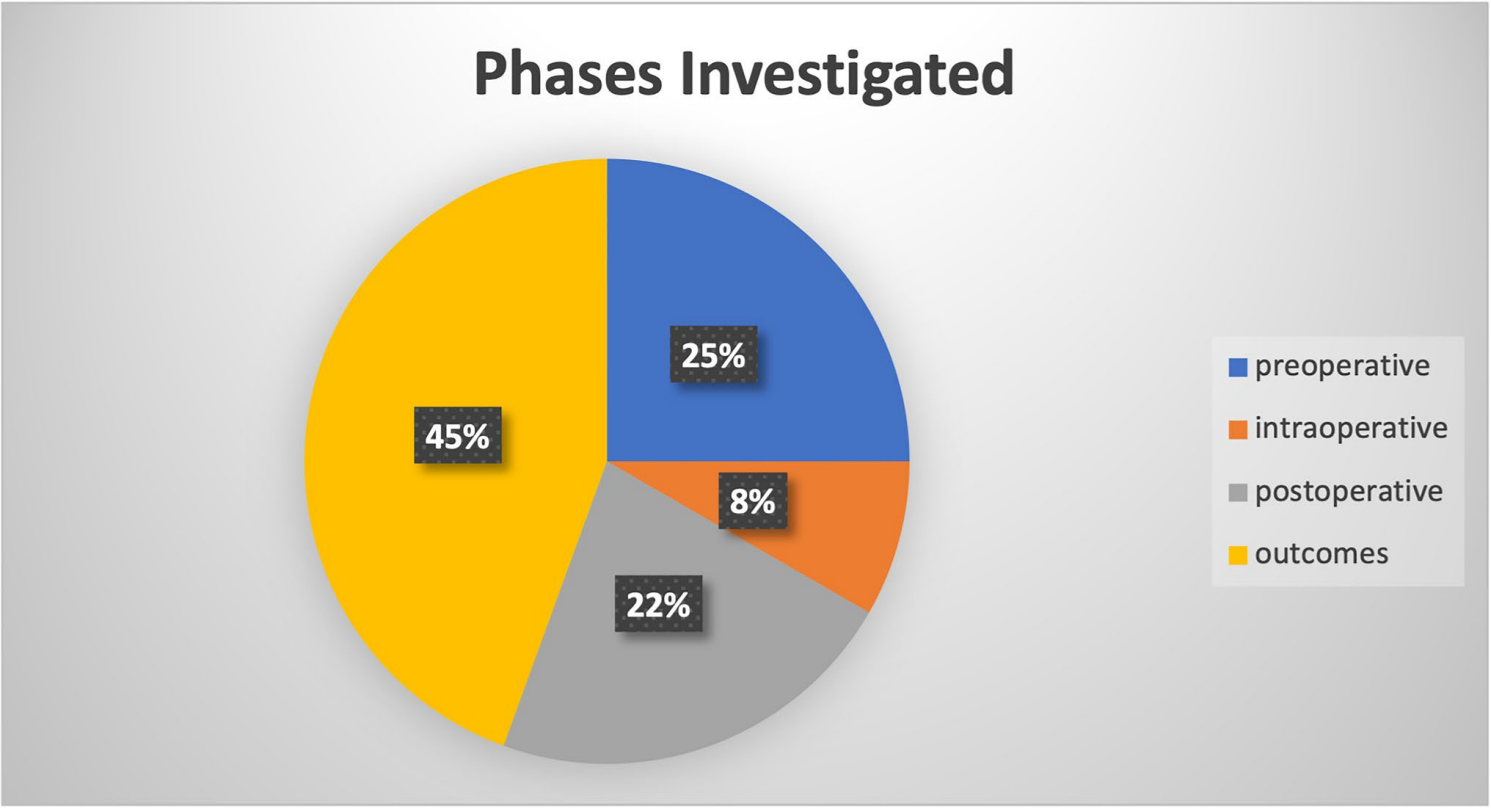
Pie chart describing the proportion of the studied involved in the review related to the specific phase of the perioperative pathway

All the included articles investigated the different steps of the management of patients with obesity; in particular, 9 studies considered the preoperative phase, 3 the intraoperative one, 8 the postoperative management and complications, while 16 studies examined the clinical outcomes (Fig. 3).

Various types of AI/ML algorithms were tested, from 1 to 19 for a single study. The most frequent.

algorithms were NN (24 articles), followed by logistic regression (LR) (16 articles), support vector machine (12 articles), random forest (5 articles), and decision tree (6 articles).

To assess the efficacy of the algorithms, multiple outcome measurements were used, specifically area under the curve (AUC) in 17 cases, accuracy in 7 cases, and sensitivity/specificity in 4 cases.

## Discussion

The management of patients suffering from obesity and candidates for a bariatric surgical procedure is highly demanding. It assumes the involvement of different specialists, in a tailored multidisciplinary approach. Many efforts have been made to facilitate physicians in accurate risk prediction, selection of the most suitable procedure, and optimization of the surgical planning, thus granting high-quality care.

In recent years, new data mining technologies are promising to be the turning point.

Along with the growing diffusion of AI-based technologies in several subspecialities of healthcare, their application in the field of BS is showing encouraging insights.

Our results are consistent with the recent findings of the scoping review by Pantelis et al. [23], as ML explored models proved effective and outperformed conventional statistical techniques.

**Fig. 4:**
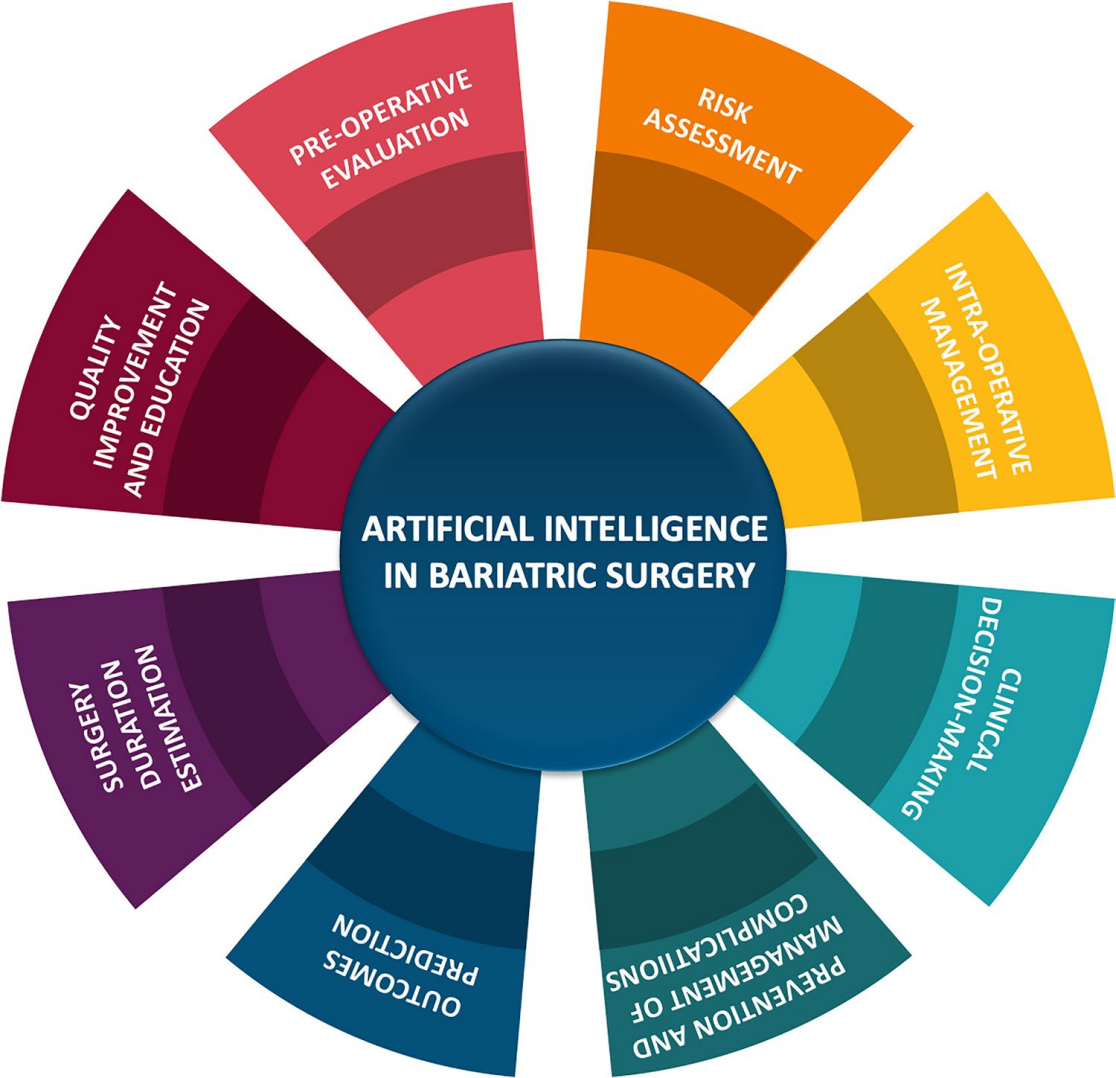
Role of artificial intelligence (AI) in bariatric surgery. AI can be used in every aspect of the perioperative path, from the presurgical assessment to the intraoperative phase, up to the postoperative management

AI algorithms have been used in each step of the clinical pathway of the patient candidates for BS, from the presurgical evaluation and intraoperative management to complications and outcome prediction (Fig. 4).

### Preoperative Evaluation and Risk-Assessment

Careful and thorough preoperative evaluation is essential in patient candidates for BS to assess their individual risk and prognosis. The aim is to find obesity-related comorbidities to identify high-risk patients and to minimize the risk of postoperative complications. AI can represent a useful tool to achieve this goal, as clearly shown from the number of studies reported (Table 1).

**Table 1:** Overview of papers about preoperative assessment included in our analysis

Author, years	Study design	Objective	Final cohort	Outcomes	Type of ML	Prediction performance
Zhou CM 2021	Retrospective single center	Prediction of difficult tracheal intubation in obese patients using six approaches from various ML fields	1015	Prediction of difficult tracheal intubation	LR, TR, RF, Gbdt, Xgbc, Gbm	Training vs testing group: LR AUC 0,68–0,70; TR AUC 0,71–0,60; RF AUC 0,92–0,58; Gbdt AUC 0,78–0,71; Xgbc AUC 0,73–0,71; Gbm AUC 0,81–0,66
Mencar C 2020	Observational multicentric	Efficacy and clinical applicability of different ML methods based on demographic information and questionnaire data to predict OSA severity	313	Prediction of obstructive sleep apnea syndrome severity	MV, NB, k-NN, Ct, RF, SVM AdaBoost-SVM, CN2 rule induction, ML, LR, k-NN, RT, SVR, AdaBoost-SVR	SVM AUC 0,65–0,61 RF AUC 0,63
Pépin JL 2020	Prospective observational single center	Evaluation of mandibular movement monitoring during sleep coupled with an automated analysis by ML for OSA diagnosis	376	OSA diagnosis	Sr RDI	Sr-RDI ≥ 5 events/h AUC 0,95; PSG-RDI ≥ 15 events/h AUC 0,93
Keshavarz Z 2020	Retrospective, single center	Development of a model for predicting OSA to select the best model to determine and screen high-risk OSA patients	231	OSA diagnosis	NN, NB, LR, KNN, SVM, RF	NN AUC 0.75; NB AUC 0,76; LR AUC 0,76; KNN AUC 0.65; SVM AUC 0.72; RF AUC 0.75
Gao WD 2019	Retrospective	Detection of OSA extracting the features of the heartbeat interval signal and the respiratory signal	N/A	OSA diagnosis	Model fusion (LR-SVM)	Sensitivity 74%, specificity 75%, accuracy 75%
Tiron R 2020	Prospective, single center	Determining of sleep and breathing patterns, and then analyzing results to track sleep-related health risks associated with sleep apnea	248	Performance of the Firefly technology as a screener for a clinical threshold of apnea hypopnea index ≥ 15	Firefly technology	ROC AUC (training 0.95, test 0.92); PR AUC (training 0.87, test 0.89)
Cheng Q 2017	Prospective, single center	Predicting pulmonary function by improved classification models with sole inputs being motion sensors from carried phones	35	To categorize patients into the correct GOLD stage	SVM	Accuracy 99%
Viswanath V 2018	Prospective, multicenter	Performing a spirometry test using only the audio data from the microphone of a standard smartphone providing automatic feedback	20505	Pulmonary function	NB, k-NN, Log Reg (L1) Log Reg (L2), RF, Gradient Boosting VGG CNN Gated-CRNN	Mel spectogram Naive Bayes precision 0.80; Mel spectogram k-NN precision 0.94; Mel spectogram Log Reg (L1) precision 0.94; Mel spectogram Log Reg (L2) precision 0.93; Mel spectogram RF precision 0.96; Mel spectogram Gradient Boosting precision 0.96; Mel spectogram VGG CNN precision 0.97; Mel spectogram Gated-CRNN precision 0.98
Assaf D 2021	Retrospective, single center	To improve preoperative diagnosis of hiatal hernia in patients candidates for BS	2482	Diagnosis of hiatal hernia	ML decision tree model	Achieving 38.5% sensitivity and 92.9% specificity, ML models increased sensitivity up to 60.2% compared to swallow study prediction

*LR* logistic regression, *TR* decision tree, *RF* random forest, *Gbdt* gradient boosting decision tree, *Xgbc* extreme gradient boosting, *Gbm* light GBM, *MV* majority vote, *NB* Naive Bayes, *k-NN* k-nearest neighbor, *Ct* classification tree, *SVM* support vector machine, *AdaBoost-SVM* adaptive boosting SVM, *ML* machine learning, *RT* regression tree, *SVR* support vector regression, *AdaBoost-SVR* adaptive boosting SVR, *Sr RDI* sunrise system-derived respiratory disturbance index, *OSA* obstructive sleep apnea, *GOLD* Global Initiative for Chronic Obstructive Lung Disease, *NN* neural network, *NB* Naïve Bayes, *ROC* receiver operating characteristics, *PR* precision recall, *ANNs* artificial neural networks, *LDA* linear discriminant analysis, *QDA* quadratic discriminant analysis, *MLP* multilayer perceptron, *AdaBoost LR* adaptive boosting LR, *CNN* convolutional neural network, *RNN* recurrent neural network, *XGBs* gradient boosting machines, *OSA* obstructive sleep apnea, *BS* bariatric surgery

For its capability to integrate a great amount of information, AI could be applicable to all preoperative patients, especially those suffering from different obesity-related comorbidities.

Airway assessment must be performed in attempt to identify possible difficult airway management. Zhou et al. explored six ML models for predicting difficult intubation in patients suffering from obesity and found three approaches that can successfully predict it. One of these, the Xgbc algorithm, has an accuracy over 80% and precision up to 100% [24].

Risk assessment of OSA is one of the aspects mostly considered for an accurate presurgical evaluation. Polysomnography (PSG) is traditionally considered an established and effective diagnostic tool providing information on the severity of OSA and the degree of sleep fragmentation. Several publications demonstrate how AI can be used to predict OSA risk. Mencar et al. tested the efficacy and clinical applicability of different ML methods based on demographic information and questionnaire data to predict OSA severity and found out that these can be useful to identify a priority level for assigning patients to the PSG test [25]. Pèpin et al. evaluated if mandibular movement monitoring during sleep coupled with an automated analysis by ML was appropriate for OSA diagnosis and found that it provided reliable performance in respiratory disturbance index [26]. Keshavarz et al. used a dataset containing self-reported variables obtained by utilizing the cross-industry standard process for data mining instruction, a methodology for medical data mining project. They found out it has a good efficacy to predict OSA and it might be a fast and cost-effective auxiliary tool [27]. Furthermore, Gao et al. studied an OSA detection algorithm based on electrocardiogram where sleep apnea-related features are obtained by extracting the time-domain and frequency-domain components of ballistocardiogram and respiratory signals over fixed time intervals. Then ML classification algorithm is used to detect OSA. This model has moderate complexity, high spatial complexity, and high sensitivity and can be used for OSA screening at home [28]. Finally, Tiron et al. presented a hybrid acoustic smartphone App that uses a signal processing technology and AI algorithms to identify sleep stages, respiration rate, snoring, and OSA patterns. It performed both reliably and accurately in the detection of clinically significant OSA, and in the estimation of apnea hypopnea index when compared to a PSG gold standard [29].

Patients with obesity often suffer from lung disfunction, in particular chronic obstructive pulmonary disease (COPD), chronic lung disease, and asthma, that can be usually detected by spirometry. It is of utmost importance to investigate these potential conditions during the preoperative evaluation, and AI proved to be equally effective in this area. Cheng et al. showed that improved classification models can accurately predict pulmonary function, with inputs being motion sensors from carried phones. The trained model perfectly computed the Global Initiative for Chronic Obstructive Lung Disease level 1, 2, and 3 [30]. Viswanath et al. analyzed and estimated the quality of smartphone spirometry efforts. They found that NN can extract more information from potentially muddled signals than traditional methods using domain-specific, expert-designed features. Indeed, it is possible to provide the necessary expert level validity feedback for smartphone-based spirometry efforts [31].

Furthermore, ML prediction models were utilized to predict preoperative hiatal hernia diagnosis. Assaf and colleagues utilized three optional ML models to improve preoperative contrast swallow study (SS) prediction, thus finding that the implementation of ML algorithms to include patient data increases the sensitivity of preoperative SS and may lower the need for hiatal exploration in a large number of patients undergoing BS [32].

ML algorithms are effective in the risk definition and are promising for the future, representing a valuable help for the clinician, increasing the efficacy of the preoperative evaluation.

However, we were not able to find any randomized trial comparing AI to standard perioperative evaluation; recently, the Wuerzburg University Hospital proposed Artificial Intelligence-augmented Perioperative Clinical Decision Support (KIPeriOP) (NCT05284227), a trial investigating a novel anesthesiologic clinical decision support (CDS) application, that integrates risk evaluation tools and updated clinical guidelines guided by artificial intelligence in the setting of preoperative anesthesiologic assessment. It will be compared to the current standard preoperative assessment workflow with participants being actual patients, and the recruitment will be starting in April 2022.

### Intraoperative Phase

There are several possible applications of AI in the intraoperative period. It could be used in the management of pharmacotheraphy, in hemodynamic optimization, in monitoring of neuromuscolar block, and of anesthesia depth [33, 34]. Despite this, its use in BS has not yet been fully explored. To our knowledge, the available literature on the use of ML in this phase is limited (Table 2).

**Table 2:** Overview of papers about intraoperative phase included in our analysis

Author, years	Study design	Objective	Final cohort	Outcomes	Type of ML	Prediction performance
Ingrande J 2020	Prospective, single center	Modeling inductionphase kinetics using a high-resolution pharmacokinetic dataset	30	Drug concentrations	4-compartment model, recirculatory model, gated recurrent unit neural network	Direct comparison of observed versus predicted concentrations
Twinanda AP 2019	Retrospective, single center	Intraoperative accurate surgery duration estimation by using only visual information from laparoscopic videos	290	Remaining surgery duration estimation	Deep Learning- a convolutional neural network and a long-short term memory network	The proposed network significantly outperforms a traditional method of estimating surgery duration without utilizing manual annotation
Hashimoto DA 2019	Retrospective, single center	To identify operative steps in laparoscopic sleeve gastrectomy	88	Automatic extraction of quantitative surgical data from operative video of laparoscopic sleeve gastrectomy	Deep Learning	SleeveNet demonstrated a mean classification accuracy of 82% ± 4% with a minimum classification accuracy of 73% and a maximum classification accuracy of 85.6%

One the most significant report concerns predicting the early distribution kinetics of propofol. In fact, the volume of distribution of drugs in patients with obesity is modified; the blood volume is increased, as well as the cardiac output, and there are alterations in the plasma transport proteins. In the study by Ingrande et al., AI has been used to manage induction-phase kinetics by means of a high-resolution pharmacokinetic dataset. A classic 4-compartment model was compared to a recirculatory model and to a gated recurrent unit NN. They found out that a recirculatory model and a gated recurrent unit artificial NN had similar performance and were both superior to a compartmental model in describing high-resolution pharmacokinetic data of propofol [35].

AI algorithms were successfully used also for accurate surgery duration estimation and quality-improvement. Twinanda et al. proposed a deep learning pipeline, referred to as RSDNet, which automatically estimates the remaining surgery duration (RSD), by using only visual information from laparoscopic videos of 120 cholecystectomies and 170 gastric bypasses. The proposed deep learning network significantly outperformed a traditional method of estimating RSD [36].

Furthermore, deep learning was utilized to automatically identify steps in laparoscopic sleeve gastrectomy from operative video with a high degree of accuracy, suggesting that advances in AI may translate to healthcare applications, future analyses of surgical cases, quality improvement, and education [37].

### Postoperative Management

#### Complications

Postoperative possible complications after BS could be divided in surgical (fistula, bleeding, herniation, anastomotic stenosis, gastric erosion, intestinal small bowel obstructions), pulmonary (deep vein thrombosis, pulmonary embolism, post-operative pneumonia), nutritional, hepato-biliary, gastrointestinal (gastric ulcers, dumping syndrome, mesenteric vein, or portal system thrombosis), and neurological (neuropathy, myopathy, encephalopathy). A tailored risk assessment could modify the perioperative management and reduce them significantly. AI can be considered a valuable help to achieve this goal (Table 3). Sheikhtaheri et al. developed a clinical decision support system to predict the early complications of one-anastomosis gastric bypass surgery. They developed different artificial neural networks (multilayer perceptron network) for prediction of 10-day, 1-month, and 3-month complications using age, body mass index (BMI), smoking status, intra-operative complications, comorbidities, laboratory tests, sonography results, and endoscopy results as factors for predicting early complications. They found out that the prediction system has a good accuracy, specificity, and sensitivity [38]. Similarly, Cao et al. aimed to find a useful ML algorithm to predict the risk for severe complication after BS. They trained and compared 29 supervised ML algorithms and observed that most of the ML algorithms showed high accuracy (> 90%) and specificity (> 90%) in both the training and test data but none of them achieved an acceptable sensitivity in the test data. Overfitting was the overwhelming problem even though some algorithms showed both high accuracy and an acceptable AUC for the training data. However, they recognized that deep learning neural networks (DLNNs) have the potential to improve the accuracy [39]. Cao et al. also published another study regarding the use of DLNNs to predict serious complications after BS. The aim was to examine whether serious postoperative complications of BS could be predicted preoperatively using DLNNs based on the information available from a national quality registry. Three supervised DLNNs were applied and compared: multilayer perceptron (MLP), convolutional neural network (CNN), and recurrent neural network (RNN). They concluded that MLP and CNN showed improved, but limited, ability for predicting the postoperative serious complications after BS, while the RNN manifested the worst performance [40]. Nudel et al. compared the ability of two ML strategies, artificial neural networks (ANNs), and gradient boosting machines (XGBs) to conventional models using LR in predicting leak and venous thromboembolism after BS. They proved that ANN and XGB outperformed traditional LR in predicting leak and could prove useful in preoperative screening [41].

**Table 3:** Overview of papers about postoperative management and complications included in our analysis

Author, years	Study design	Objective	Final cohort	Outcomes	Type of ML	Prediction performance
Sheikhtaheri A 2019	Retrospective, multicenter	Predicting the early complications of one-anastomosis gastric bypass	1509	Complications incidence	ANNs	Accuracy, specificity, sensitivity: 10-day prediction system 98.4%, 98.6%, 98.3%;1-month system 96%, 93%, and 98.4%; 3-month system 89.3%, 86.6%, 91.5%
Cao Y 2019	Retrospective, multicenter	Predicting the risk for severe complication after BS	37811	Complications incidence	LR, LDA, QDA, TR, KNN, SVM, MLP, NN, AdaBoost LR, bagging LDA, bagging QDA, RF, extremely randomized trees, AdaBoost Extra trees, gradient RT, AdaBoost Gradient trees, bagging KNN, AdaBoost SVM, bagging MLP	Best gradient RT and bagging MLP AUC 0.58
Cao Y 2020	Retrospective, multicenter	Exploring whether serious postoperative complications of bariatric surgery recorded in a national quality registry can be predicted preoperatively using deep learning methods	44061	Complications incidence	MLP, CNN, RNN	AUC ≤ 0.6
Nudel J 2021	Retrospective, multicenter	Predicting leak and VTE after BS	436807	Leak and VTE incidence	ANN, XGBs	ANN AUC 0.75; XGBs AUC 0.70
Wise ES 2020	Retrospective, multicenter	To optimize the prediction of the composite endpoint of 30-day readmission, reoperation, reintervention, or mortality, after laparoscopic sleeve gastrectomy	101721	30-day morbidity and mortality prediction after bariatric surgery	LR and ANN	ANN AUROC = 0.581 compared to LR AUROC = 0.572 in the training set
Razzaghi T 2019	Retrospective, multicenter	To identify risks/outcomes associated with BS	11636	Risk-prediction	NB, Radial Basis Function Neural Network, k-NN, SVM, and LR	The combination of a suitable feature selection method with ensemble learning methods equipped with Oversampling (SMOTE) method can achieve higher performance metrics
Cruz MR 2014	Retrospective, single center	To validate a computerized intelligent decision support system that suggests nutritional diagnoses of patients submitted to BS	60	Nutritional monitoring of patients undergoing BS	Bayesian network	The system sensibility and specificity were 95.0%
Liew PL 2007	Retrospective, single center	To compare the predictive accuracy of LR and ANN with respect to the clinicopathologic features of gallbladder disease among obese patients	117	Prediction of gallbladder disease	LR and ANN	The average correct classification rate of ANNs was higher than that of the traditional logistic regression approach (97.14% versus 88.2%). Besides, ANNs also had a lower Type II error when compared with logistic regression

*LR* logistic regression, *TR* decision tree, *RF* random forest, *Gbdt* gradient boosting decision tree, *Xgbc* extreme gradient boosting, *Gbm* light GBM, *MV* majority vote, *NB* Naive Bayes, *k-NN* k-nearest neighbor, *Ct* classification tree, *SVM* support vector machine, *AdaBoost-SVM* adaptive boosting SVM, *ML* machine learning, *RT* regression tree, *SVR* support vector regression, *AdaBoost-SVR* adaptive boosting SVR, *Sr RDI* sunrise system-derived respiratory disturbance index, *OSA* obstructive sleep apnea, *GOLD* Global Initiative for Chronic Obstructive Lung Disease, *NN* neural network, *NB* Naïve Bayes, *ROC* receiver operating characteristics, *PR* precision recall, *ANNs* artificial neural networks, *LDA* linear discriminant analysis, *QDA* quadratic discriminant analysis, *MLP* multilayer perceptron, *AdaBoost LR* adaptive boosting LR, *CNN* convolutional nearal network, *RNN* recurrent neural network, *XGBs* gradient boosting machines, *BS* bariatric surgery, *VTE* venous thromboembolism

Comparable results about the effectiveness of AI algorithms to predict morbidity and mortality after BS were obtained by other groups. Recently, Wise et al. used an ANN model to optimize the prediction of 30-day readmission, reoperation, reintervention, or mortality after laparoscopic sleeve gastrectomy, compared to standard LR modeling [42]. Similarly, the results by Razzaghi and colleagues demonstrated the potential of ML tools as clinical decision support in identifying risks/outcomes associated with BS and their effectiveness in reducing the surgery complications and in improving patient care [43].

Furthermore, AI models can help and facilitate physicians in the prevention and management of metabolic complications after BS. In fact, a computerized intelligent decision-making support system for nutritional diagnoses was specifically developed and validated, thus assisting health professionals in the nutritional monitoring of patients submitted to BS [44].

ANN might be as well a useful tool to predict the risk factors and prevalence of gallbladder disease and gallstone development in patients suffering from obesity on the basis of multiple variables related to laboratory and pathological features [45].

In conclusion, ML methods can offer clinically meaningful improvements in risk stratification, even for uncommon events that are difficult to predict using traditional statistical method.

#### Outcomes

AI based models found wide space in clinical outcome prediction after BS, especially regarding weight loss, obesity related diseases remission, and postoperative quality of life (Table 4).

**Table 4:** Overview of papers about clinical outcomes included in our analysis

Author, years	Study design	Objective	Final cohort	Outcomes	Type of ML	Prediction performance
Zhang W 2021	Prospective, single center	To predict optimal weight loss 6 months after BS	37	Classification of patients with optimal and suboptimal weight loss at 6 months post BS	Siamese-kNN, LR, SVM	The Siamese-KNN achieved an accuracy of 83.78% and AUC of 0.84
Modaresnezhad M 2019	Retrospective, multicenter	To enable a large reduction in dimensionality of the data and to allow for fast and efficient application of data mining techniques to large clinical datasets	120,000	Prediction of BS outcomes	TR, regression, and NN	The rule-based semantic approach for reducing data dimensionality was highly effective in reducing the volume of the data and the time needed to run the analysis. The reduced model performs as well as the full model
Celik S 2020	Retrospective, single center	To verify the dependence of weight loss on sleeve coefficients and to forecast the weight loss	63	Prediction of weight loss after laparoscopic sleeve gastrectomy	SVM, neural network Bayesian regularization	Levenberg–Marquardt and Bayesian regularization are the most suitable algorithms. Error intervals were smaller for Bayesian regularization algorithm and are broader for Levenberg-Marquardt algorithm
Wise ES 2016	Retrospective, single center	To devise a web-based tool to predict excess BMI loss after laparoscopic RYGB by identification of independent preoperative predictors	647	Prediction of excess weight loss after laparoscopic RYGB	ANN	AUC of ANN for the training set and validation set were 0.78 ± 0.03 and 0.83 ± 0.04, respectively
Piaggi P 2010	Prospective, single center	To build a statistical model based on psychological and physical data to predict weight loss in patients treated by LAGB	172	Weight loss prediction in obese candidates to LAGB	ANN	Nonlinear model resulted to be better at data fitting (36% vs. 10% variance explained, respectively) and provided more reliable parameters for accuracy and mis-classification rates (70% and 30% vs. 66% and 34%, respectively)
Lee YC 2007	Prospective, single center	To evaluate weight reduction after BS using information available during the initial preoperative assessment	249	Prediction of weight reduction	LR and ANN	The overall predictive accuracy of ANN is higher than logistic regression in the prediction of successful weight reduction
Dimeglio C 2020	Retrospective, single center	To analyze the postoperative weight trajectories and to identify “curve families” for early prediction of weight regain	795	Prediction of weight evolution	Hierarchical cluster analysis	Classification with reference trajectories produced an overall rate of correct classification of more than 93%
van Loon SLM 2020	Retrospective, single center	To objectively quantify the metabolic health status of patients after BS	1595	The Metabolic Health Index can quantify the improvement in the metabolic health status of treated bariatric patients	LR	The index reflects severity of comorbidity, enabling objective assessment of a bariatric patient’s metabolic health state, regardless day of sampling and surgery type
Johnston SS 2019	Retrospective, multicenter	To develop a predictive model of antihyperglycemic medication cessation after metabolic surgery	16527	No antihyperglycemic medication treatment from 365 to 730 days after metabolic surgery	LR	The model possessed good internal discriminative accuracy (AUC = 0.778) and transportability (external AUC = 0.759)
Lee WJ 2012	Retrospective, single center	To examine the efficacy of surgically induced weight loss on diabetes remission	88	Prediction of diabetes remission	LR and ANN	The average correct classification rate of logistic regression was 85.9%, The average correct classification rate of the ANN model was 90.4%
Aminian A 2020	Retrospective, single center	Constructing and internally validating prediction models to estimate the risk of long-term end-organ complications and mortality in patients with type 2 diabetes and obesity	2287	End organ complication detection	Regression, RF	Surgery versus usual care: all-cause mortality (AUC 0.79 and 0.81), coronary artery events (AUC 0.66 and 0.67), heart failure (AUC 0.73 and 0.75), and nephropathy (AUC 0.73 and 0.76)
Aron-Wisnewsky J 2017	Retrospective, single center	To develop an improved scoring system for predicting diabetes remission following RYGB	352	Prediction of diabetes remission 1 year post BS	Multivariate logistic regression	Ad-DiaRem displayed improved AUROC and predictive accuracy compared with DiaRem (0.911 vs 0.856 and 0.841 vs 0.789, respectively; *p* = 0.03)
Debédat J 2018	Retrospective, single center	To develop an improved scoring system for predicting long-term diabetes remission following RYGB	175	Prediction of long-term diabetes remission	Fully corrective binning	The score was accurate AUROC = 90%; accuracy = 85% at predicting 5-years diabetes remission
Pedersen HK 2016	Retrospective, multicenter	To stratify individuals based on clinical and genomic factors that determine their diabetic response to surgery, and to identify factors that have an important role in this response	457	Discrimination between patients with and without surgery-induced diabetes remission	ANN	Accuracy = 74%, AUC = 0.81
Cao Y 2019	Retrospective, multicenter	To predict 5-year health-related quality of life after bariatric surgery based on the available preoperative information	6687	Long-term quality of life prediction in patients after BS	CNN	The CNN model showed an overwhelming advantage in predicting all the health-related quality of life measures
Cao Y 2020	Retrospective, multicenter	To find better methods for predicting prognosis and provide evidence for patient management after BS	6542	Long-term outcome prediction in patients after BS	BN, CNN, Multivariate LR	BN showed excellent predictive ability for 5-year type 2 diabetes and dyslipidemia (AUC = 0.942 and 0.917, respectively), good ability for 5-year hypertension and sleep apnea syndrome (AUC = 0.891 and 0.834, respectively), and fair ability for 5-year depression (AUC = 0.750)

*LR* logistic regression, *TR* decision tree, *RF* random forest, *Gbdt* gradient boosting decision tree, *Xgbc* extreme gradient boosting, *Gbm* light GBM, *MV* majority vote, *NB* Naive Bayes, *k-NN* k-nearest neighbor, *Ct* classification tree, *SVM* support vector machine, *AdaBoost-SVM* adaptive boosting SVM, *ML* machine learning, *RT* regression tree, *SVR* support vector regression, *AdaBoost-SVR* adaptive boosting SVR, *Sr RDI* sunrise system-derived respiratory disturbance index, *OSA* obstructive sleep apnea, *GOLD* Global Initiative for Chronic Obstructive Lung Disease, *NN* neural network, *NB* Naïve Bayes, *ROC* receiver operating characteristics, *PR* precision recall, *ANNs* artificial neural networks, *LDA* linear discriminant analysis, *QDA* quadratic discriminant analysis, *MLP* multilayer perceptron, *AdaBoost LR* adaptive boosting LR, *CNN* convolutional neural network, *RNN* recurrent neural network, *XGBs* gradient boosting machines, *BS* bariatric surgery, *BMI* body mass index, *RYGB* Roux-en-Y gastric bypass, *LAGB* = laparoscopic adjustable gastric banding

Effectiveness of BS, by means of the forecast of weight loss, is one the fields in which AI was mainly implemented, thus assisting in personalized diagnosis for treatment of obesity, and in the selection of the best candidates for surgery [45–48].

ANN modeling was used to provide an optimized estimate of expected postoperative weight loss at 6 and 12 months after laparoscopic Roux-en-Y gastric bypass (LRYGB) using only known preoperative patient variables [49]. Similarly, ANN models were successfully applied for prediction of weight loss in women with obesity treated by laparoscopic adjustable gastric banding (LAGB) [50], and laparoscopic one anastomosis gastric bypass (LOAGB) [51].

In the recent paper by Dimeglio et al., a hierarchical cluster analysis was used to identify four profiles of weight trajectories associated with clinical expertise. Interestingly, the authors reported that patients who were the most successful were those who lost weight regularly, and patients who lost the least had difficulties in the initial phase or had a secondary weight regain [52].

Moving to prediction of obesity related comorbidities improvement, ML was applied to develop an ordinal LR model, using 4 clinical and 32 laboratory input variables, and the output was then mathematically transformed into a continuous score for intuitive interpretation, ranging from 1 to 6. In analogy with BMI as index for weight, the Metabolic Health Index (MHI) is developed as objective quantification of metabolic health status, to objectively express improvement of comorbidity [53].

We found several papers investigating the application of AI in the prediction of diabetes remission, proving that it could be helpful for personalized management of individuals with obesity and diabetes candidates for BS, contributing ultimately to precision medicine.

The application of ML techniques to real-world healthcare data can yield useful predictive models to assist the selection of patients more responsive to surgery-induced diabetes remission [54, 55].

Aminian et al. aimed to construct and validate prediction models to estimate the risk of long-term end-organ complications and mortality in patients affected by type 2 diabetes and obesity. The prediction models were programmed to construct user-friendly web-based and smartphone applications of individualized diabetes complications (IDC) risk scores for clinical use. They analyzed all-cause mortality, coronary artery events, heart failure, and nephropathy, showing that these major adverse cardiovascular events are predictable outcomes in patients with diabetes and obesity who undergo metabolic surgery or received diabetes care. They concluded that the IDC Risk Scores can provide personalized evidence-based risk information for patients with type 2 diabetes and obesity about future cardiovascular outcomes and mortality with and without metabolic surgery based on their status of obesity, diabetes, and related cardiometabolic conditions [56].

Aron-Wisnewsky et al. described the development of the Ad-DiaRem scoring system for predicting diabetes remission following RYGB in individuals with obesity and type 2 diabetes. They demonstrated the ability of the score to better separate between individuals predicted to achieve remission and those who will not, and the score improved predictive performance over the traditional scoring system [57]. The same group validated the score even with a long-term follow-up of 5 years [58]. Furthermore, similar findings on the discrimination of patients with and without surgery-induced diabetes remission were reported in the study by Pedersen et al. [59].

Bayesian networks provide useful tools for predicting long-term health-related quality of life in patients after BS, based on their preoperative health and disease status, and outperformed CNN and multivariable LR, in the recent paper by Cao et al. [60, 61].

We were not able to find any paper exploiting the use of AI in the determination of the most proper surgical procedure for each patient. Nevertheless, we do believe that, in the near future, the use of data mining technologies will be extremely useful to select out the best operation for a given patient. In fact, in past years, the number of surgical cases of each bariatric center did not allow the analysis to adequately answer this key topic of BS. Currently, with the introduction of electronic medical records and the availability of large datasets from different centers, we will enter a new era of clinical research, possibly giving a solution to open questions.

## Future Perspectives and Limitations

As reported for other medical subspecialties, along with the application of data mining technologies, the use of telemedicine in the field of BS could be one the explorable area in the future. To date, available literature is limited [62].

Despite the promising results of the use of AI-models in each phase of the perioperative management of the patients with obesity, some concern remains about legal and ethical aspects.

In fact, these technologies assume the availability of high-quality datasets, and the collection and utilization of medical data should fulfill regulation criteria, as the ones of the General Data Protection and Regulation (GDPR), that has been issued by the European Union [63, 64]. Unfortunately, most of the time, these existing regulations do not specifically deepen the issue of new technologies and even less they do not give precise and transparent legal instruction for the processing of health data by AI techniques.

Furthermore, separate consensus guidelines are needed to report inherent studies, thus increasing transparency and accuracy of results [65].

Moreover, as clearly inferable from the geographical distribution of the studied included in our analysis, the accessibility to AI and ML methods is not equally available worldwide, contributing to the exacerbation of social inequities. This is a phenomenon opposite to that for which they were born, that is, the smoothing out of differences. In the near future, it will therefore be important to create international networks capable of bypassing national limits and favoring technological access. Similarly, the non-equal access to these technologies, due to substantial costs and economic discrepancy, is still a concern. Indeed, to date, the use of AI could be considered more expensive and not cost-effective, when compared to standard evaluation. Actually, with the continuous application, over time and on large scale, the use of AI-based technologies could lead to a maximization of resources, especially in the accurate and proper plan of surgical procedures, and thus in the optimization of operating rooms efficiency.

Finally, all healthcare professionals should be properly educated and trained on these techniques, granting the full development of AI-instruments potential [66]. In fact, the equipment and instrumentation that are commonly available make AI-tools widely accessible, but the education and culture of the involved physicians can guarantee the specific competence, necessary to extrapolate the maximum use for patients’ care.

## Conclusions

AI algorithms have been used in each step of the perioperative path of the patient candidates for BS, from the presurgical evaluation and risk-assessment to postoperative complications and outcomes prediction.

ML models are promising encouraging results helping physicians in the decision-making process in the management of the patients with obesity candidates for BS, thus improving the quality of care and contributing to the goal of precision medicine.

Nevertheless, a number of legal and ethical hurdles remain to be overcome before these methods can be really integrated in common practice.
